# Analgesia With Epidural Anesthesia in Lower-Leg Necrotizing Fasciitis: A Case Report

**DOI:** 10.7759/cureus.80314

**Published:** 2025-03-09

**Authors:** Tatsuya Fujihara, Yasuyuki Ochi, Takumi Gobara, Tetsuro Nikai

**Affiliations:** 1 Anesthesiology, Shimane University Hospital, Izumo, JPN; 2 Dermatology, Shimane University Hospital, Izumo, JPN

**Keywords:** analgesia, critical care outreach team, debridement, epidural anesthesia, necrotizing fasciitis

## Abstract

Appropriate methods have not been established despite the necessity of analgesia for debridement in cases of necrotizing fasciitis.

A 45-year-old woman was hospitalized for necrotizing fasciitis due to *Streptococcus pyogenes*. Rapid debridement and antibiotic treatment enabled the patient’s quick discharge from the intensive care unit. However, after discharge, she began experiencing considerable pain in the left lower leg, which hindered adequate debridement, resulting in the persistence of necrotic tissue. Epidural anesthesia was administered to alleviate pain and enable debridement.

Epidural anesthesia was effective as analgesia in this case, facilitating infection control through debridement and alleviating procedural pain.

## Introduction

Necrotizing infections often involve tissue damage. In particular, necrotizing fasciitis occurring secondary to *Streptococcus pyogenes *infection can develop at sites of blunt trauma, such as bruises, in approximately 50% of cases [1-3]. Necrotizing fasciitis, including necrosis of fascial tissue, typically progresses within hours, and the mortality rate associated with necrotizing infections is high. Treatment involves prompt debridement and antibiotic administration [1-5]. For infection control in cases of necrotizing fasciitis, multiple debridement procedures may be required at times, and opioids such as morphine are commonly used as analgesics for these procedures; however, achieving adequate pain relief can be challenging [6].

Herein, we report the case of a patient with subacute necrotizing fasciitis in whom effective pain control was achieved using epidural anesthesia after inadequate pain management with fentanyl and ketamine resulted in nausea and vomiting.

## Case presentation

**Figure 1:**
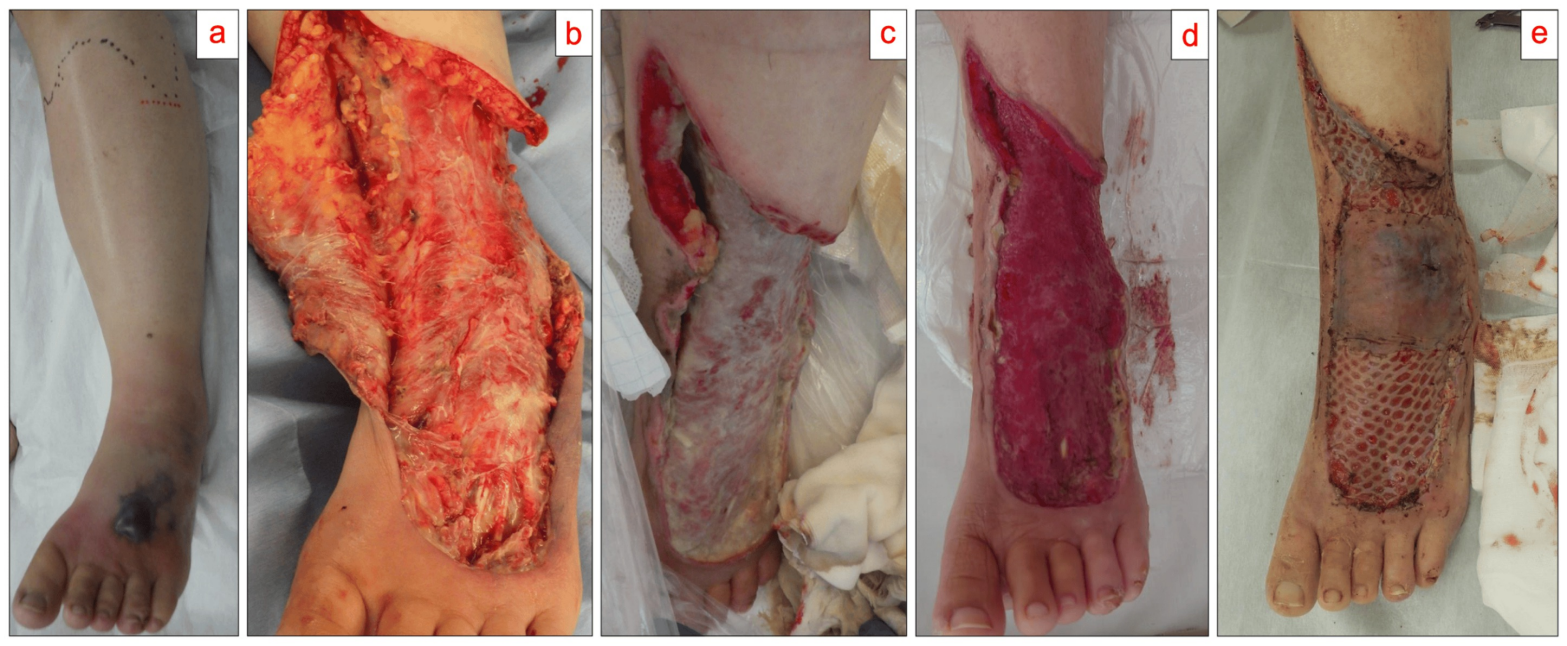
Course of findings in a case of necrotizing fasciitis in the left lower leg. (a) On admission, a necrotized blister is observed on the dorsum of the left foot. The finger test is positive. (b) Immediately after debridement, dishwater-like drainage is observed. (c) On the eighth day of hospitalization, necrotic tissue remains. (d) Four days after epidural anesthesia is performed, analgesia is achieved, and necrotic tissue disappears with adequate debridement. (e) On the fifth day after skin graft implantation, the growth is good, and no recurrence of infection is noted.

A 45-year-old Japanese woman, 158.9 cm, 54.8 kg, who had undergone surgery for uterine fibroids three years prior, which included laparoscopic hysterectomy and salpingo-oophorectomy, was admitted to the hospital with necrotizing fasciitis triggered by blunt trauma to the dorsum of her left foot (Figure 1a).

On the day of admission, debridement was performed as soon as possible, and meropenem and clindamycin were administered (Figure 1b). Owing to the need for fluid resuscitation and noradrenaline for circulatory management, the patient was admitted to the intensive care unit (ICU) after the procedure. The day after admission, gram-positive cocci, which were identified as *S. pyogenes* (group A *Streptococcus *(GAS)) by matrix-assisted laser desorption/ionization time-of-flight mass spectrometry using VITEK^®️^ MS Knowledge Base v3.2 (bioMérieux, France), were detected in 2/2 sets of blood cultures. *S. pyogenes* was also detected in cultures of pus and tissue samples collected during debridement, confirming the diagnosis of necrotizing fasciitis caused by *S. pyogenes*. In vitro susceptibility testing revealed that it had the following minimum inhibitory concentrations (MICs): penicillin G, ≦ 0.06 μg/mL; ampicillin, ≦ 0.12 μg/mL; cefotaxime, ≦ 0.06 μg/mL; ceftriaxone, ≦ 0.25; erythromycin, ≦ 0.25; clindamycin, ≦ 0.25; vancomycin, ≦ 0.25. Consequently, treatment was de-escalated from meropenem to ampicillin based on the susceptibility results. Following debridement and antibiotic treatment, the patient’s circulatory status gradually stabilized, allowing for the discontinuation of noradrenaline four days after admission. During the patient’s ICU stay, fentanyl or ketamine was administered for pain relief during daily wound debridement. However, she experienced severe nausea despite the administration of antiemetics such as ondansetron and metoclopramide, and it was determined that the patient could not tolerate both drugs well. Anticipating continued procedures in the general ward, pentazocine, acetaminophen, and celecoxib were administered to manage the pain. Upon confirming the absence of nausea, the patient was discharged from the ICU on the fifth day of hospitalization.

However, during a critical care outreach team (CCOT) round on the eighth day of hospitalization, the patient reported pain at rest as well as during the procedure. The corresponding scores for her pain on the numeric rating scale (NRS) were 5/10 and 8/10, respectively. Although no expansion of the infection site was observed, residual necrotic tissue was still locally present, necessitating continuous debridement (Figure 1c). Following the suggestion of the anesthesiologist participating in the CCOT round, epidural anesthesia was planned. Before the insertion of the epidural catheter, we confirmed that there was no infection at the puncture site and no coagulation or hemostasis abnormalities, and the patient was not using any antiplatelet or anticoagulant agents (platelet count: 33.9 × 10^4^/μL, activated partial thromboplastin time (APTT): 26.4 seconds, and prothrombin time (PT)-international normalized ratio (INR): 1.02). We administered epidural anesthesia at the L4/5 level on the ninth day of hospitalization. The procedure was initiated, and the NRS score during the procedure decreased to 1/10. After the procedure, a patient-controlled epidural analgesia regimen of 0.17% levobupivacaine (0.25% levobupivacaine 200 mL mixed with 100 mL of normal saline) was initiated at a rate of 2 mL/h, which reduced the pain at rest to approximately 0/10 on the NRS. In the general ward, it was decided that 4 mL of 2% mepivacaine would be administered as a bolus before each procedure, as this additional dose provided adequate analgesia and maintained stable vital signs during the wound procedure following the initial induction of epidural anesthesia. The management of the epidural catheter was handled by the CCOT, ensuring that there were no complications such as catheter malposition or paralysis. The epidural catheter was removed on the eighth day after placement. Once adequate control of the infection site was achieved (Figure 1d), a skin grafting procedure was performed on the 23rd day of hospitalization, and the skin graft was well tolerated (Figure 1e). The patient was discharged after being hospitalized for 43 days.

## Discussion

Herein, we described our experience with a case of necrotizing fasciitis in which effective pain control was achieved using epidural anesthesia. Effective pain relief provided by epidural anesthesia facilitated optimal management of the infectious source through debridement.

An increase in invasive GAS infections has been reported in Japan [7]. Necrotizing fasciitis, particularly *S. pyogenes*-induced necrotizing fasciitis, is triggered by blunt trauma in approximately 50% of cases, is associated with a high mortality rate, and necessitates early surgical intervention [1-3]. For necrotizing fasciitis, early and complete debridement has the most significant impact on the ultimate outcome. Opioids remain the mainstay for pain management in most ICU settings [8]. Morphine is the most commonly used analgesic and is used in nearly 90% of necrotizing fasciitis cases owing to extensive and multiple debridement procedures required [2,3]. However, pain management has been reported to be ineffective in 13-29% of these cases [6]. Other pain management options, such as ketamine, acetaminophen, nonsteroidal anti-inflammatory drugs, gabapentin, and oral opioids, have been utilized by palliative care teams employing a multimodal pain approach [4,9]. Although there have been reports of a serratus plane block without an indwelling catheter performed for necrotizing fasciitis of the axilla [10], regional anesthesia in necrotizing fasciitis remains a challenge. This is because, similarly, in the acute phase of necrotizing fasciitis, extensive and multiple debridement procedures are necessary [6]. It is necessary to determine the extent of necrotizing fasciitis to avoid complications such as regional anesthetic infection. Furthermore, while necrotizing fasciitis develops in the limbs in many cases, the site of onset varies, and infection has also been noted in the perineum, buttocks, torso, and head and neck region [3]. This diversity of the affected areas may also complicate pain management.

In this case, pain management was challenging owing to nausea and vomiting induced by fentanyl and ketamine, which hindered adequate debridement. We attempted pain management with opioids in the hyperacute phase of necrotizing fasciitis. Epidural catheter use tends to be avoided in patients with an untreated systemic infection [11]. However, the possibility that epidural anesthesia can be safely performed even in the presence of bacteremia has also been discussed [12]. Therefore, we administered effective antibiotics for more than a week and after confirming that no signs of infection at the puncture site and no coagulation abnormalities were present, we performed epidural anesthesia because it allows for stable and easily controlled continuous administration to achieve complete debridement. Additionally, the CCOT round facilitated the evaluation of complications. If careful evaluation shows that the necrotizing fasciitis is localized with no coagulation abnormalities, risks like epidural hematoma can be minimized. If the affected area can be managed with epidural or peripheral nerve blocks, these techniques may be valuable for pain management. This is particularly relevant as invasive GAS infections continue to increase.

## Conclusions

We noted pain complaints from a patient with necrotizing fasciitis in the left lower leg during a round conducted by the CCOT. Epidural anesthesia was effective for analgesia in this case, facilitating infection control through debridement and alleviating procedural pain. Regional anesthesia techniques may be valuable options for pain management for necrotizing fasciitis.
